# α-Synuclein Oligomer Detection with Aptamer Switch on Reduced Graphene Oxide Electrode

**DOI:** 10.3390/nano10050832

**Published:** 2020-04-27

**Authors:** Seung Joo Jang, Chang-Seuk Lee, Tae Hyun Kim

**Affiliations:** Department of Chemistry, Soonchunhyang University, Asan 31538, Korea; seen813@sch.ac.kr (S.J.J.); eriklee0329@sch.ac.kr (C.-S.L.)

**Keywords:** Parkinson’s disease, α-synuclein, reduced graphene oxide (rGO), aptamer, aptasensor

## Abstract

Protein aggregation of alpha-synuclein (α-Syn) is implicated in Parkinson’s disease (PD), and, thus, α-Syn aggregates are a potentially promising candidate biomarker for PD diagnosis. Here, we describe a simple and sensitive electrochemical sensor to monitor the aggregation of α-Syn for early PD diagnosis. The sensor utilizes methylene blue (MB)-tagged aptamer (Apt) adsorbed on electrochemically reduced graphene oxide (ERGO) by π–π stacking. The binding of α-Syn oligomer to the Apt induces desorption of the Apt from the ERGO surface, which leads to the electrochemical signal change. The resulting sensor allowed the highly sensitive and selective detection of α-Syn oligomer according to the voltammetric change. Under optimized conditions, the linear range of detection was observed to be from 1 fM to 1 nM of the α-Syn oligomer and the limit of detection (LOD) was estimated to be 0.64 fM based on S/N = 3. The sensor also showed good reproducibility and stability, enabling real sample analysis of the α-Syn oligomer in human blood serum. With its ultrasensitivity and good performance for α-Syn oligomer detection, the sensor provides one promising tool for the early diagnosis of PD.

## 1. Introduction

Parkinson’s disease (PD) is the second most common neurodegenerative disorder that causes a person to lose control over some body functions [1,2]. PD can be clinically characterized by motor symptoms such as bradykinesia (slowness of movement), dyskinesia (involuntary muscle movement), increased muscle rigidity, resting tremors, and postural instability, along with non-motor symptoms such as depression, psychosis, and cognitive impairment [3,4,5,6]. Recent estimates indicate that approximately 50,000 people receive a PD diagnosis each year, and the prevalence of PD is anticipated to increase to approximately 770,000 by the year 2040 in the United States [7,8,9]. Therefore, there has been an increase in demand for early PD diagnosis to enable early neuroprotective treatment leading to better clinical results and eventually better quality of life in PD patients [10,11,12]. Current findings have shown that the pathogenesis of PD may be contributed by the development of Lewy bodies, abnormal protein aggregations at the synaptic terminal of midbrain dopaminergic neurons. α-synuclein (α-Syn) is known to be the main underlying protein responsible for Lewy bodies [13,14]. The degradation system of α-Syn is presumed to be defective in pathological conditions, making monomers of α-Syn aggregate into oligomers, which are regarded as the harmful species associated with neuronal death in the early stages of PD [15]. α-Syn oligomer levels are typically 0.01 (0.7 fM)–0.03 (2.1 fM) pg/mL for a healthy person in blood plasma, while excess levels of α-Syn oligomer in PD patents are 70.8 to 0.14 pM [16]. To date, various efforts have been made to develop new techniques to sensitively detect α-Syn oligomers as a biomarker for PD diagnosis. These techniques include methods based on mass spectrometry [17,18], capillary electrophoresis [19,20], nuclear magnetic resonance spectroscopy [21,22], fluorescence spectroscopy [23,24], and surface plasmon resonance [25]. Many of these techniques require costly and complex instruments and suffer from time-consuming and multistep sample preparation. Meanwhile, electrochemical methods are highly advantageous due to their properties of low-cost, simple detection mechanism, ease of miniaturization, and high sensitivity and selectivity. To develop an electrochemical sensor with good performance, amplified signal generation and efficient modification of the electrode with the recognition element are crucial factors [26,27,28]. With the advancement in bioelectrochemical technology and nanomaterials, the electrochemical aptasensor based on target-recognizing aptamer appears to be the most promising approach [29,30]. The electrochemical aptasensor is a versatile sensing platform employing aptamers as recognition elements, which are attached at one end to an electrode and modified at the other end with a redox reporter. The binding of the target to the aptamer alters the redox efficiency of the reporter, thereby producing an electrochemical signal change [31,32,33].

**Scheme 1:**
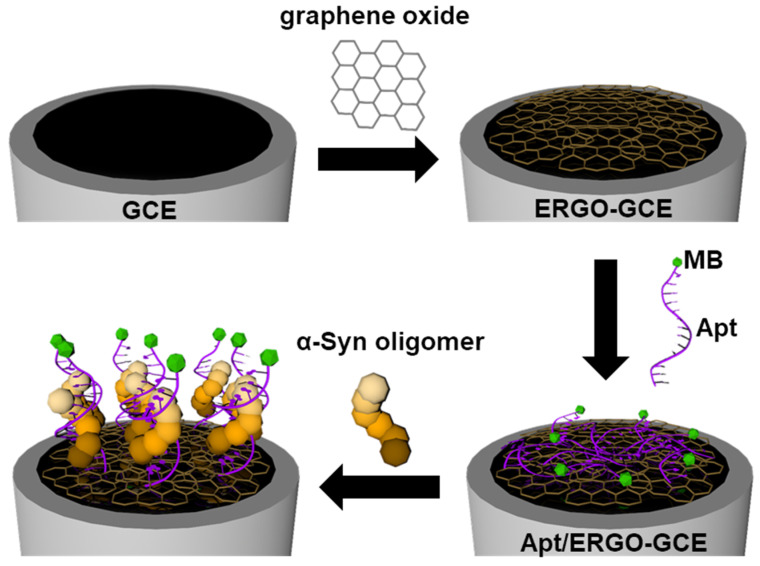
Illustration depicting the fabrication of aptamer (Apt)/electrochemically reduced graphene oxide-glassy carbon electrode (ERGO-GCE) for the detection of α-synuclein (α-Syn) oligomer.

Herein, we propose a simple and sensitive electrochemical aptasensor for α-Syn oligomers based on an electrochemically reduced graphene oxide (ERGO) electrode modified with α-Syn oligomer-recognizing aptamer (Apt). Scheme 1 shows the fabrication and detection principle of the electrochemical aptasensor, denoted as Apt/ERGO-glassy carbon electrode (GCE), for α-Syn oligomers. ERGO was first formed and modified on a glassy carbon electrode (GCE) by the electrochemical reduction of GO. Then, Apt tagged by methylene blue (MB) was coated on the ERGO-GCE via π–π stacking interaction. The binding of α-Syn oligomer to Apt might induce the conformational change of the Apt and its subsequent detachment from the ERGO surface, which can therefore generate an electrochemical signal change. It is noted that the efficient enrichment of electrocatalytic ERGO on the electrode and direct adsorption of Apt onto the ERGO may generate amplified electrochemical signals for the ultrasensitive detection of α-Syn oligomer.

By using the Apt/ERGO-GCE, we could sensitively detect α-Syn oligomers with a limit of detection (LOD) of 0.64 fM based on S/N = 3. The proposed sensor showed a good selectivity and response for α-Syn oligomers over the other interfering species. Along with its good reproducibility and stability, this indicates the suitability of the sensor for the detection of α-Syn oligomer in real samples. As a proof of concept, we could indeed detect α-Syn oligomer in human blood serum.

## 2. Materials and Methods

### 2.1. Materials and Instruments

Apt was purchased from BIONEER Corporation (Daejeon, Korea) and the sequence is 5′-Methylene Blue-TTTTTGGTGGCTGGAGGGGGCGCGAACG-3′, which was taken from the literature [25,34]. Graphite powder, potassium permanganate (KMnO_4_), hydrogen peroxide (H_2_O_2_), sodium nitrate (NaNO_3_), sulfuric acid (H_2_SO_4_), phosphate buffer saline (PBS, pH 7.4, 0.01 M), K_3_Fe(CN)_6_, tris-buffered saline (TBS; 0.1 M Tris-HCl, 0.3 M NaCl, pH 7.6), glucose oxidase (GOx), myoglobin (Myo), immunoglobulin G (IgG), human serum albumin (HSA), bovine serum albumin (BSA), and α-synuclein human were purchased from Sigma-Aldrich Co. (St. Louis, MO, USA). All chemicals were of analytical grade and used without further purification. Deionized (DI) water (18 MΩ) purified by a MilliQ system (Millipore Korea, Co., Ltd., Seoul, South Korea) was used throughout the experiments.

Electrochemical measurements were carried out on a CHI 660D electrochemical analyzer (CH Instruments, Inc., Austin, TX, USA). Electrochemical impedance spectroscopy (EIS) studies were performed in a 0.1 M TBS buffer solution containing 5.0 mM K_3_Fe(CN)_6_. EIS was recorded between 10^5^ and 10^−2^ Hz at a sinusoidal voltage perturbation of 5 mV amplitude. A conventional three-electrode cell was used, including a bare GCE (BAS MF-2012, diameter 3.0 mm), ERGO-GCE, and Apt/ERGO-GCE as working electrodes, a platinum wire (BAS MW-1032) as the counter electrode, and a Ag/AgCl electrode (BAS MF-2052, RE-5B) as the reference electrode. Stock solutions of α-Syn oligomer and potential interfering proteins (Myo, IgG, GOx, BSA, HSA) with various concentrations were prepared using 0.1 M TBS buffer. For the preparation of α-synuclein in molar concentration, 14.4 kDa of α-synuclein was transformed to a molar concentration with the equation in a previously report [35]. The wettability of electrodes was measured with a contact angle analyzer (Phoenix mini, SEO Co., Ltd., Suwon, Korea). The Raman spectrum was recorded with an EnSpectr R532 Raman spectrometer (Enhanced Spectrometry, Inc., San Jose, CA, USA). Fourier-transform infrared spectroscopy (FTIR) analysis was carried out with a NICOLET iS10 system (Thermo Scientific Korea Ltd., Seoul, South Korea). Scanning electron microscopy (SEM) measurements were done using a JSM 5600 LV system (JEOL, Tokyo, Japan).

### 2.2. Preparation of Apt/ERGO-GCE

ERGO-GCE was fabricated by the similar procedures reported previously [29,30]. GO was acquired from graphite powders by the modified-Hummers method [36]. As-prepared GO (0.3 mg·mL^−1^ dispersed in 10 mM PBS buffer, pH 7.4) was ultrasonicated to form homogeneous yellow-brown GO for 1 h (Sonics Vibracell VC750, Sonics&Materials Inc. Newton, CT, USA). GCE was polished first with aqueous slurries of 1.0, 0.3, and 0.05 μm Al_2_O_3_ powders, followed by sonication for 10 min in ethanol and DI water (1:1, ethanol:DI water) mixed solution. After that, GCE was further cleaned by electrochemical cleansing in 0.25 mM H_2_SO_4_ solution with a cyclic voltammetry (CV) scanning range of −1.0 to 1.0 V (vs. Ag/AgCl) with a scan rate of 50 mV/s for 20 cycles. The GCE was decorated with ERGO by electrochemical deposition, which was synthesized by CV from −1.5 to 0.8 V (vs. Ag/AgCl) at a scan rate of 10 mV/s for 3 cycles with 10 mM PBS containing 0.3 mg/mL of GO (Figure S1A). The resulting ERGO-modified GCE (ERGO-GCE) was washed with DI water and dried with nitrogen gas. To immobilize Apt onto ERGO-GCE, the electrode was immersed in stirred solution of 1 μM Apt (in 0.1 M pH 7.6 TBS solution) for 40 min. After immobilization, the fabricated electrode was washed with DI water. Before use, the electrodes were stored in 0.1 M TBS buffer.

## 3. Results and Discussion

### 3.1. Charaterization of Apt/ERGO-GCE

**Figure 1:**
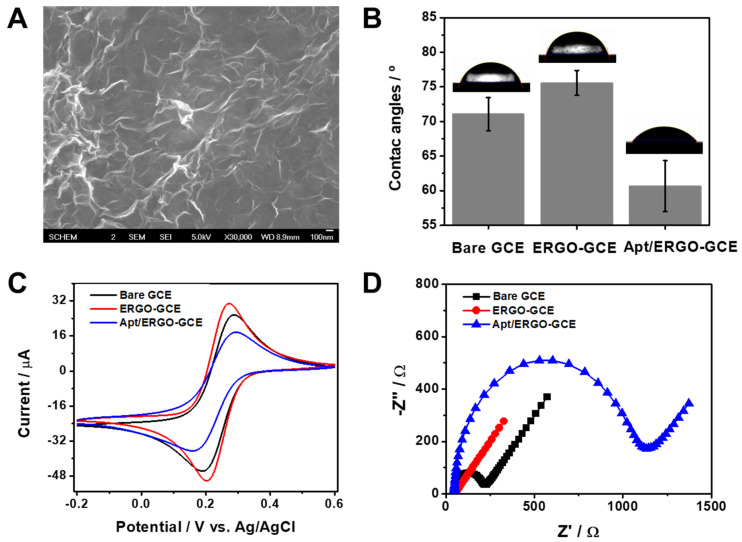
(**A**) Scanning electron microscopy (SEM) image of ERGO-GCE. (**B**) Contact angle data of water droplets on GCE, ERGO-GCE, and Apt/ERGO-GCE. (**C**) Cyclic voltammetry (CV) curves recorded at a scan rate of 10 mV·s^−1^ and (**D**) Nyquist plots of GCE, ERGO-GCE, and Apt/ERGO-GCE in 0.1 M tris-buffered saline (TBS) buffer containing 5.0 mM [Fe(CN)_6_]^3−^.

To fabricate ERGO-GCE, we synthesized and deposited ERGO on GCE with a different number of CV cycles in the presence of GO in PBS. Figure S1A represents a typical CV curve showing irreversible cathodic waves around −0.44 and −0.73 V, which decrease in subsequent scans, corresponding to the reduction process of oxygen functionalities in GO. To optimize the electrodeposition and formation of ERGO on the GCE surface, the active surface areas of the ERGO-GCEs prepared with different numbers of CV cycles were calculated using the Randles–Sevcik equation from the CV curves obtained using 0.1 M KCl containing 5 mM [Fe(CN)_6_]^3−^ at different scan rates (Figure S1B). As shown in Figure S1C, the active surface area increased with the increasing number of CV cycles and saturated at three cycles of CV, and was thus chosen as the optimum number of CV cycles. The formation of ERGO on the GCE surface was characterized by SEM. The SEM image of ERGO-GCE (Figure 1A) shows the formation and deposition of a wrinkled ERGO on GCE, unlike that of bare GCE (Figure S1D) showing a relatively smooth surface. Raman and FT-IR spectra confirm that noticeable structural changes occurred during the electrochemical reduction of GO to ERGO. In Figure S2A, GO and ERGO exhibited the typical D and G band around 1340 and 1600 cm^−1^, respectively. However, the intensity ratio of D and G bands (*I_D_/I_G_*) increased from 1.1 to 1.7 after the reduction of GO to ERGO. This suggests that the average size of the sp^2^ carbon network decreased by the removal of oxygen functional groups. Moreover, a novel peak in the Raman spectra of ERGO appeared at 2672 cm^−1^ corresponding to the stacking nature of graphene layers. Figure S2B shows FT-IR spectra of GO and ERGO. While GO exhibited peaks assigned to oxygen functionalities such as hydroxyl, epoxide, and carbonyl functional groups, ERGO showed the disappearance or substantial decrease of those peaks. Along with CV and SEM data, this indicates the successful fabrication of ERGO-GCE with a large electroactive area and stable coating. Subsequently, we functionalized as-synthesized ERGO-GCE with Apt via π–π stacking to construct an Apt/ERGO-GCE-based electrochemical aptasensor for an α-Syn oligomer. To examine the stepwise fabrication process of the Apt/ERGO-GCE, we compared the wettability and the electron transfer kinetics of GCE, ERGO-GCE, and Apt/ERGO-GCE by studying their contact angles of water droplets on the surface, and their voltammetric and impedimetric behaviors using [Fe(CN)_6_]^3−^ as a redox probe. As shown in Figure 1B, contact angle data for water droplets on the ERGO-GCE showed more hydrophobic surface (76° ± 1.6°) than that of bare GCE (71° ± 2.1°). The further modification of ERGO-GCE with Apt, however, led to the decrease in the contact angle (61° ± 3.2°), indicating increasing wettability of the generated surface due to the negatively charged structure of Apt with a sugar-phosphate backbone. Figure 1C,D show CV curves and EIS spectra of GCE, ERGO-GCE, and Apt/ERGO-GCE in 0.1 M TBS (pH 7.6) containing 5.0 mM [Fe(CN)_6_]^3−^. As shown in Figure 1C, ERGO-GCE exhibited higher peak currents (*i_p_*) and smaller peak potential separations (*ΔE_p_*) than those of bare GCE, meaning the high electron transfer kinetics in ERGO-GCE owing to excellent conductivity and the large surface area of ERGO. However, the modification of ERGO-GCE with Apt resulted in an *i_p_* decrease and *ΔE_p_* increase, due to the repulsive forces between the [Fe(CN)_6_]^3−^ and the negatively charged phosphate ions formed on the electrode surface by the Apt coating. The EIS data were in full accordance with voltammetric results (Figure 1D). Charge transfer resistance (*R_ct_*), reflecting the electron transfer kinetics of the redox probe, displayed a lower value for ERGO-GCE than that for bare GCE, indicating the better conductivity from the nature of graphene materials. Then, the modification of the electrode with Apt caused a large increase in *R_ct_* value, implying that the negatively charged Apt was immobilized on the ERGO-GCE and the phosphates in Apt repelled the negatively charged [Fe(CN)_6_]^3−^ ions. Along with contact angle data, these electrochemical results confirm that all fabrication steps for Apt/ERGO-GCE were successfully carried out.

### 3.2. Sensing Principle and Optimization of Experimental Parameters

**Figure 2:**
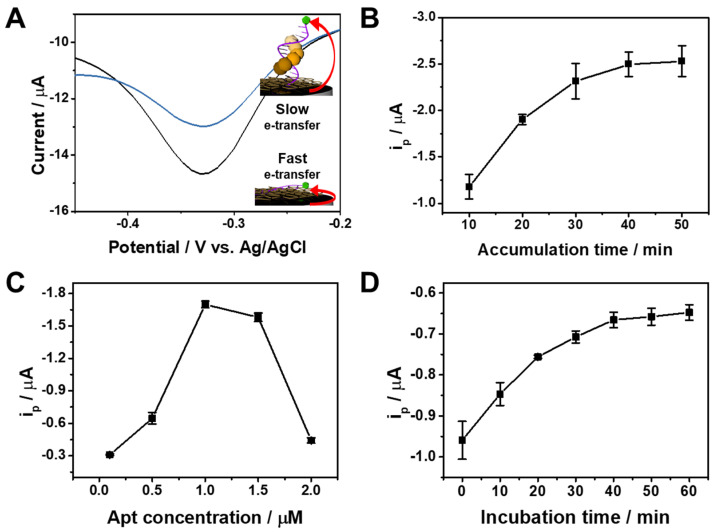
(**A**) Differential pulse voltammetry (DPV) curves of Apt/ERGO-GCE in the absence and presence of α-Syn oligomer in 0.1 M TBS. Effect of (**B**) the accumulation times ([Apt] = 1 μM) and (**C**) Apt concentration (accumulation time = 40 min) for Apt immobilization on *i_p_* in 0.1 M TBS buffer. (**D**) Effect of incubation time for α-Syn oligomer binding on *i_p_* in 0.1 M TBS buffer. [α-Syn oligomer] = 1 nM, [Apt] = 1 μM, Accumulation time = 40 min, Room temperature.

In order to clarify the sensing mechanism for α-Syn oligomer, we performed EIS experiments of Apt/ERGO-GCE in the presence of increasing concentrations of α-Syn oligomer. As shown in Figure S3A, the R_ct_ value increased with the concentration of α-Syn oligomer. This indicates binding between Apt and α-Syn oligomer on the ERGO surface, which leads to the impediment of electron transfer between [Fe(CN)_6_]^3−^ and the electrode due to the bulky structure of the α-Syn oligomer-Apt complex on the ERGO-GCE (Figure S3B). The binding complex also hinders the electron transfer between the MB tag and the electrode due to the far proximity of the MB tag from the electrode surface as a result of the binding of α-Syn oligomer to Apt. With the use of the electroactive MB tag in Apt as an electrochemical indicator, voltammetric analysis using Apt/ERGO-GCE is possible. To test if the proposed sensing principle works well, the sensor was investigated by CV and differential pulse voltammetry (DPV) measurements. Figure S4 shows the typical CV curves of Apt/ERGO-GCE upon the addition of increasing concentrations of α-Syn oligomer (0–1 nM) in 0.1 M TBS buffer. Apt/ERGO-GCE exhibited an irreversible cathodic current peak at about −0.60 V and anodic current shoulder at about 0 V, which decreased upon the addition of increasing concentrations of α-Syn oligomer. However, the redox peaks of Apt/ERGO-GCE and their variations upon the addition of α-Syn oligomer were not distinct enough for sensing. To get a better resolution of redox peaks, DPV was performed. Figure 2A shows the typical DPV curves of Apt/ERGO-GCE in the absence and presence of α-Syn oligomer in 0.1 M TBS (pH 7.6). A strong reduction peak current (*i_p_*) appeared at −0.33 V, which corresponds to the electrochemical reduction of the MB tag, while the addition of α-Syn oligomer resulted in a smaller peak current during the reduction of the MB tag in Apt/ERGO-GCE. This indicates that α-Syn oligomer is bound to the Apt, following the desorption of the Apt from the surface of ERGO-GCE, which hampers the electron transfer between the MB tag and the electrode. To achieve the best electrochemical performance of Apt/ERGO-GCE, we examined the effect of accumulation time and concentration for Apt immobilization on the voltammetric response of the electrode in 0.1 M TBS buffer (Figure 2B,C). To optimize the accumulation time for immobilization of the Apt on ERGO-GCE, we examined the effect of the immersion time of ERGO-GCE into the Apt solution on *i_p_*. The voltammetric response of Apt/ERGO-GCE exhibited an increase in *i_p_* with increasing time of Apt accumulation in the range of 10−40 min, and afterward, *i_p_* was saturated (Figure 2B). Thus, 40 min could be enough for the Apt immobilization. In the case of Apt concentration, the *i_p_* value showed its maximum at 1 μM (Figure 2C). Therefore, 1 μM was selected in the experiment. The influence of incubation time for α-Syn oligomer binding on voltammetric response was also examined, as shown in Figure 2D. The *i_p_* value increased with increasing incubation time, and then tended to saturate gradually around 40 min, and was thus chosen as the incubation time for α-Syn oligomer binding.

### 3.3. Electrochemical Detection of α-Syn Oligomer with Apt/ERGO-GCE

**Figure 3:**
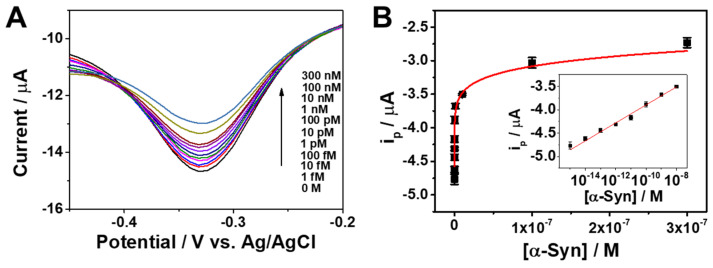
(**A**) Differential pulse voltammetry (DPV) curves of Apt/ERGO-GCE upon the addition of different concentrations of α-Syn oligomer in 0.1 M TBS buffer. The applied potential was scanned from 0.1 to −0.5 V (vs. Ag/AgCl) with an amplitude of 0.05 V, pulse width of 0.05 s, and pulse time of 0.5 s. (**B**) Corresponding calibration plot for *i_p_* versus α-Syn oligomer concentration. The inset is the linear relationship between *i_p_* and the logarithmic concentration of α-Syn oligomer.

Under optimal conditions, we evaluated the analytical performance of the Apt/ERGO-GCE-based sensor for the detection of α-Syn oligomer. Figure 3A shows DPV curves of Apt/ERGO-GCE with different concentrations of α-Syn oligomer in a 0.1 M TBS buffer. The *i_p_* value of the sensor gradually decreased with increasing concentration of α-Syn oligomer from 1 fM to 300 nM. The corresponding calibration plot is displayed in Figure 3B. The Figure 3B inset shows the linear relationship between *i_p_* and the logarithmic concentration of α-Syn oligomer in the range from 1 fM to 1 nM, which can be expressed as *i_p_* (μA) = −2.0 × 10^−6^ + 1.9 × 10^−7^ logC (M) (*R*^2^ = 0.99). The limit of detection (LOD) was calculated to be 0.64 fM based on an S/N ratio of 3. The analytical performance of the proposed sensing strategy was compared to other electrochemical sensing methods using different electrodes in terms of the linear range and LOD (Table 1). This represents that the proposed sensor exhibits excellent analytical performance of the sensing strategy for α-Syn oligomer detection.

**Figure 4:**
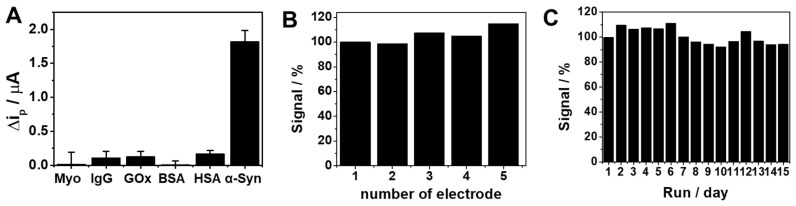
(**A**) Voltammetric responses (*Δi_p_*) of Apt/ERGO-GCE in the absence and presence of α-Syn oligomer and potential interfering proteins (myoglobin (Myo), immunoglobulin G (IgG), glucose oxidase (GOx), bovine serum albumin (BSA), human serum albumin (HSA)) in 0.1 M TBS buffer. Concentrations of α-Syn oligomer and other proteins are 100 pM and 10 nM, respectively. (**B**) Reproducibility of the sensor for five different modified electrodes. (**C**) Stability of the sensor in 15 days under atmosphere conditions.

We further investigated the analytical performance including selectivity, reproducibility, and stability for the practical application of the proposed sensing strategy (Figure 4). Figure 4A shows the *i_p_* changes (*Δi_p_*) of the sensor upon the addition of α-Syn oligomer or potential interfering proteins such as Myo, IgG, GOx, BSA, and HSA. The addition of each interfering protein caused a negligible response, whereas the addition of α-Syn oligomer showed a significant change, representing the good selectivity of our sensing strategy. The sensor also showed good reproducibility, which was investigated in 0.1 M TBS buffer with five electrodes prepared independently. Figure 4B shows similar voltammetric responses of the electrodes with acceptable relative standard deviation (RSD = 6.2%). In addition, to evaluate the stability of the sensor, the electrode was stored under atmospheric conditions for 15 days. As shown in Figure 4C, the sensor preserved 94% of its primary response with an RSD value of 6.3%, indicating acceptable long-term stability. The proposed biosensor is, however, not adequate for multi-shot sensing, due to its relatively slow response (~40 min) for now. For the continuous assay of α-Syn oligomer, further improvement will be necessary for the reaction time.

### 3.4. Detection of α-Syn Oligomer in Real Sample

As a proof of concept, the applicability of the proposed sensing system in a real sample analysis for α-Syn oligomer detection was examined with human serum. The human blood serum with electrolyte solution was spiked with different concentrations of α-Syn oligomer and the measurement was performed using the standard addition method. As shown in Table 2, the results showed good recovery and relative standard deviation (RSD) values, representing the possibility of practical use of the proposed sensing strategy in the real sample. However, we did not perform the analysis of different α-Syn protein forms, such as monomer and fibril, which can be present in the real application. In addition, as α-Syn is a pre-synaptic protein, real samples can be difficult to find and could require complex sample pre-treatment. For these reasons, the proposed sensing system cannot be exploited in practical bio-clinical applications for the moment. Further studies are needed to monitor the aggregation of α-Syn for practical clinical applications.

## 4. Conclusions

We have described a facile fabrication of an ultrasensitive electrochemical aptasensor for α-Syn oligomer. The sensor utilizes a sensitive ERGO-GCE directly interfaced with Apt, which is employed to instantaneously transduce the binding activities between α-Syn oligomer and Apt. The direct interfacing of Apt and its instantaneous response to α-Syn oligomer allows a high sensitivity and selectivity. In our sensing strategy, the presence of α-Syn oligomer causes desorption of the Apt from the ERGO-GCE surface, leading to the sluggish electron transfer between the MB tag and the electrode. The resulting Apt/ERGO-GCE exhibited excellent analytical performances including sensitivity, selectivity, reproducibility, and stability, indicating the feasibility of the practical application for α-Syn oligomer detection. Our sensor may provide a powerful platform for the detection of α-Syn oligomer in the diagnosis of individuals.

## Figures and Tables

**Table 1:** The comparison of the sensing performance of the proposed sensing strategy with other electrochemical sensing methods for α-Syn oligomer.

Electrode Materials	Method	Linear Range	LOD	Reference
{HRP-Ab2-GNPs}/Ab1/-SYN/Th/PAMAMAu/o-ABA/GCE	CV	1.6 pM–16 nM	1 pM	[1]
Apt-CS/α-Syn oligomer/Exo I/TdT/Au	DPV	60 pM–150 nM	10 pM	[34]
Cystamine/GO/Au	EIS	5 pM–1 nM	1.2 pM	[37]
TiO_2_/Au/Ab1/α-Syn/{Ab2-Au-GOx}	PEC	4.2 pM–8.3 nM	2.3 pM	[38]
AuNPs/PGA/EDC-NHS/Anti-α-Syn alpha/BSA/ITO	SWV	0.3 pM–0.2 nM	9.3 fM	[39]
Apt/ERGO-GCE	DPV	1 fM–1 nM	0.64 fM	This work

**Table 2:** α-Syn oligomer detection in human serum for the real sample application of Apt/ERGO-GCE.

Sample	Amount Added (M)	Amount Found (M)	RSD (%) (*n* = 3)	Recovery (%)
Human serum	1 × 10^−12^	0.98 × 10^−12^	1.15	98.5
	1 × 10^−9^	0.99 × 10^−9^	2.39	99.7

## Supplementary Materials

The following are available online at https://www.mdpi.com/2079-4991/10/5/832/s1, Figure S1: CV curves recorded at GCE in 10 mM PBS buffer solution (pH 7.4) containing 0.3 mg mL^−1^ GO at a scan rate of 10 mV for 3 cycles of CV. B. Relationship between peak current and square root of scan rate for oxidation of ERGO-GCEs with different number of CV cycles (1, 2, 3, 4, 5) in 0.1 M KCl containing 5 mM [Fe(CN)_6_]^3−^. C. Plot of active surface areas of ERGO-GCEs with different number of CV cycles (1, 2, 3, 4, 5) in 0.1 M KCl containing 5 mM [Fe(CN)_6_]^3−^. D. SEM image of bare GCE. Figure S2: A. Raman spectra of GO and ERGO. B. FT-IR spectra of GO and ERGO. Figure S3: A. Nyquist plots for the Apt/ERGO-GCE in different concentrations of α-syn oligomer (0 M, 1 fM, 1 pM, and 1 nM) in 0.1 M TBS buffer containing 5.0 mM [Fe(CN)_6_]^3−^. B. Plausible sensing mechanism of Apt/ERGO-GCE for detection of α-syn oligomer. Figure S4: CV curves of Apt/ERGO-GCE at a scan rate of 50 mV s^−1^ upon the addition of different concentrations of α-Syn oligomer (0–1 nM) in 0.1 M TBS buffer.
