# Supplementary material for: α-Synuclein Oligomer Detection with Aptamer Switch on Reduced Graphene Oxide Electrode
Source: Nanomaterials (Basel). 2020 Apr 27;10(5):832. doi: 10.3390/nano10050832 (PMC7711764; doi:10.3390/nano10050832)
Supplement: Supplementary file 1 [file nanomaterials-10-00832-s001.pdf]

nanomaterials

MDPI

Supplementary Materials

α-synuclein oligomer detection with aptamer switch on reduced graphene oxide electrode

Seung Joo Jang†, Chang-Seuk Lee†, and Tae Hyun Kim*

Department of Chemistry, Soonchunhyang University, Asan 31538, Korea; seen813@sch.ac.kr (S.J.J.) ; eriklee0329@sch.ac.kr (C.-S.L.)

* Correspondence: thkim@sch.ac.kr; Tel.: +82-41-530-4722

† These authors contributed equally to this work.

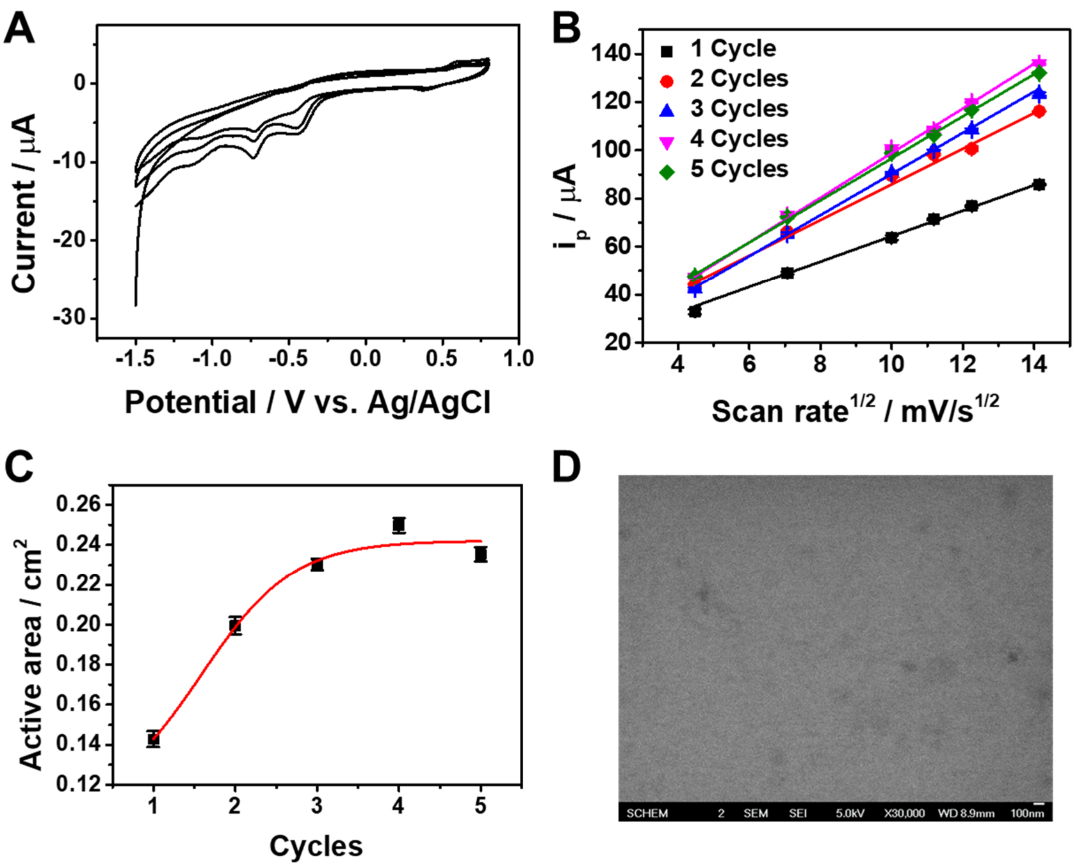

Figure S1. A. CV curves recorded at GCE in 10 mM PBS buffer solution (pH 7.4) containing 0.3 mg mL^{-1} GO at a scan rate of 10 mV for 3 cycles of CV. **B.** Relationship between peak current and square root of scan rate for oxidation of ERGO-GCEs with different number of CV cycles (1, 2, 3, 4, 5) in 0.1 M KCl containing 5 mM $[Fe(CN)_6]^{3-}$. **C.** Plot of active surface areas of ERGO-GCEs with different number of CV cycles (1, 2, 3, 4, 5) in 0.1 M KCl containing 5 mM $[Fe(CN)_6]^{3-}$. **D.** SEM image of bare GCE.

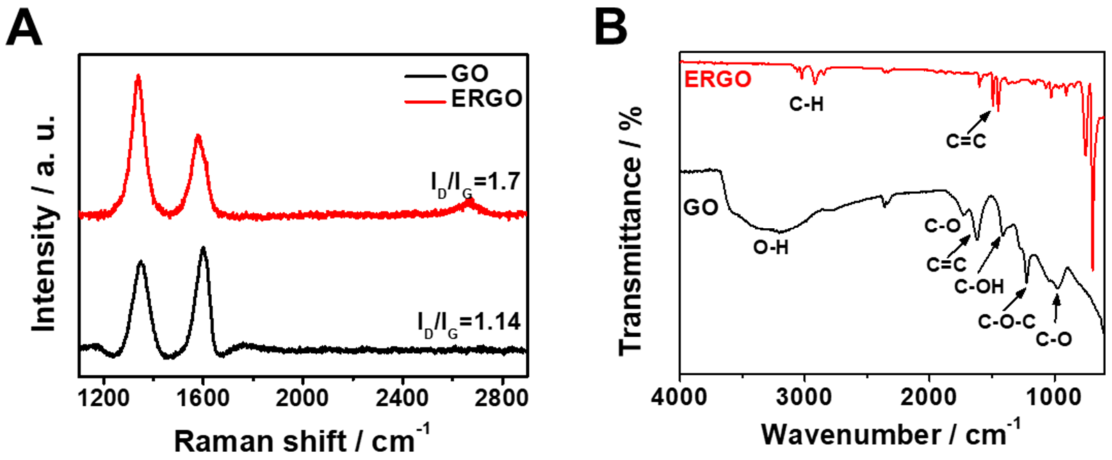

Figure S2. A. Raman spectra of GO and ERGO. **B.** FT-IR spectra of GO and ERGO.

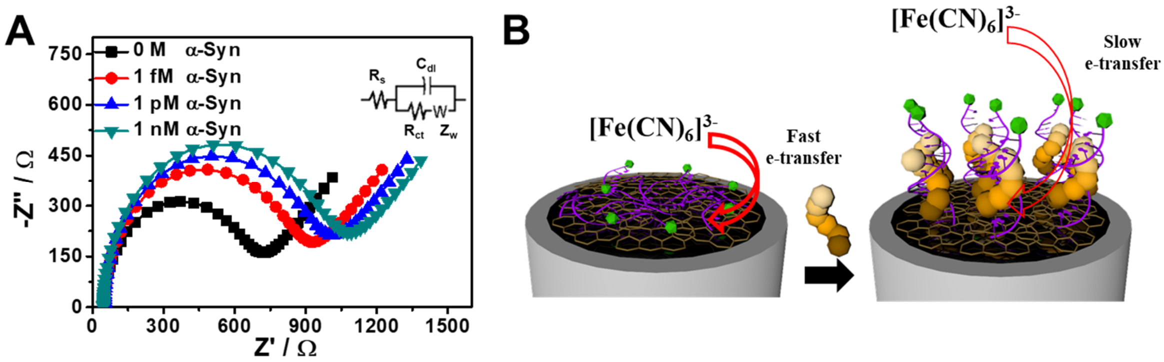

Figure S3. A. Nyquist plots for the Apt/ERGO-GCE in different concentrations of α-syn oligomer (0 M, 1 fM, 1 pM, and 1 nM) in 0.1 M TBS buffer containing 5.0 mM $[Fe(CN)_6]^{3-}$. **B.** Plausible sensing mechanism of Apt/ERGO-GCE for detection of α-syn oligomer.

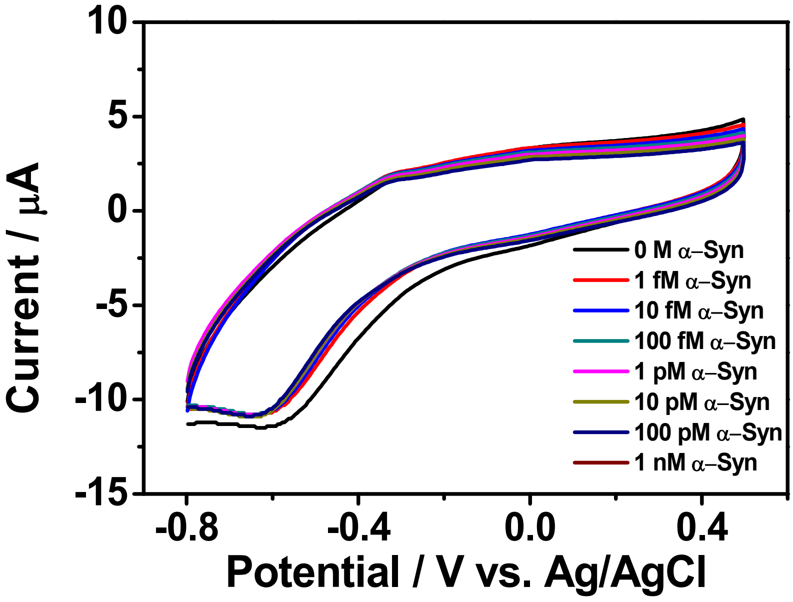

Figure S4. CV curves of Apt/ERGO-GCE at a scan rate of 50 mV s^{-1} upon the addition of different concentrations of α-Syn oligomer (0 – 1 nM) in 0.1 M TBS buffer.
